# Isolation of 4000 SARS-CoV-2 shows that contagiousness is associated with viral load, not vaccine or symptomatic status

**DOI:** 10.1080/22221751.2021.2008776

**Published:** 2021-12-02

**Authors:** Celine Boschi, Sarah Aherfi, Linda Houhamdi, Philippe Colson, Didier Raoult, Bernard La Scola

**Affiliations:** a Microbes, Evolution, Phylogeny and Infection (MEPHI), UM63, Institut de Recherche pour le Développement (IRD), Assistance Publique - Hôpitaux de Marseille (AP-HM), Aix-Marseille University, Marseille, France; b IHU Méditerranée Infection, Marseille, France

**Keywords:** SARS-CoV-2, culture, delta variant, symptomatology, vaccination

## Abstract

Culture inoculation of 6722 nasopharyngeal samples since February 2020 allowed isolation of 3637 SARS-CoV-2 and confirmed that isolation rate is correlated to viral load, regardless symptomatology or vaccination status. Moreover, the delta variant is associated with higher viral loads and therefore higher rates of viral isolation, explaining its greater contagiousness.

Since February 2020 our BSL3 laboratory has been involved in massive SARS-CoV-2 isolation [1,2]. As a consequence, we early established a clear correlation between viral load and isolation of the virus in cell culture*,* which is an indirect marker of patient contagiousness. It was later suggested that asymptomatic SARS-CoV-2-infected patients or vaccinated infected patients would have a lower viral load and/or would be less contagious [3,4]. This prompted us to analyse the 6731 nasopharyngeal samples with a *Ct* < 35 at time of diagnosis inoculated in our BSL3 laboratory until July 2021 under previously described conditions [1,2]. Vaccinated patients were defined as those diagnosed ≥ 15 days after their first injection of any of the four vaccines available in Europe (https://ec.europa.eu/info/live-work-travel-eu/coronavirus-response/safe-covid-19-vaccines-europeans_fr). Then, we tested if the predominant SARS-CoV-2 variant at time of diagnosis was associated with a different *Ct* and culture positivity rate. We studied four periods: (i) February–September 2020 (Wuhan-Hu-1 close virus derivatives (French original viruses)), (ii) October–December 2020 (predominance of 20A.EU2 variant, in 1684/2135 (79%) of the samples with viral genotype available); (iii) January–June 2020 (predominance of alpha variant; 9060/14,495 (63%) samples with viral genotype available); and (iv) July 2021 (delta variant, 1932/2031 (95%) samples with viral genotype available). Statistical analysis was performed on GraphPad prism 5.03 using One-way Anova or Mann–Whitney tests.

We could isolate 3637 (54%) SARS-CoV-2 from the 6722 patients’s samples inoculated (mean age ± standard deviation: 60.5 years old ± 21 years old). Culture positivity was inversely proportional to *Ct* at diagnosis as *Ct* was significantly lower for patients with a positive than negative culture (mean ± standard deviation: 23.2 ± 4.83 versus 28.3 ± 4.9, respectively; *p* < 0.0001) (Figure 1(a)). Symptomatic or asymptomatic status was known for 3761 and 543 patients, respectively. Mean *Ct* was significantly lower in asymptomatic than symptomatic patients (23.1 ± 5.9 versus 26.1 ± 5.4, respectively; *p* < 0.0001) (Figure 1(a)). Regarding culture isolation, it was positive for 50% (1882/3761) of symptomatic patients compared to 69% (372/543) of asymptomatic patients (*p* < 0.0001) (Figure 1(b)). Since January 2021, we inoculated 309 samples from vaccinated patients and 433 samples from unvaccinated. *Ct* at diagnosis was significantly lower for vaccinated patients than unvaccinated patients (21.5 ± 4.5 versus 23.4 ± 5.4, respectively) (Figure 1(a)) (*p* < 0.0001). We isolated 80% (249/309) of SARS-CoV-2 among vaccinated patients versus 66% (287/433) among unvaccinated patients (*p* < 0.0001) (Figure 1(b)). For the 150 vaccinated patients for whom this information was available, 134 (89%) were symptomatic and 16 (11%) were asymptomatic (Figure 1(c)). We were able to isolate 74% and 63% of strains among the vaccinated patients symptomatic and asymptomatic, respectively (NS). For the 209 unvaccinated patients for whom this information was available, 167 (80%) were symptomatic and 42 (20%) were asymptomatic. The proportion of symptomatic patients was statistically different from that among vaccinated patients (*p *= 0.008) (Figure 1(c)). We were able to isolate 65% and 72% of strains among the unvaccinated patients symptomatic and asymptomatic, respectively (NS). Finally, there was no significant difference between mean *Ct* of the periods with the French original SARS-CoV-2 and during which the 20A.EU2 variant predominated (25.6 ± 5.5 versus 25.7 ± 5.5 respectively). In contrast, mean *Ct* was significantly lower for periods during which the alpha (22.6 ± 5.2) then delta variants (19.7 ± 3.4) predominated than for the two former periods, and between January and June than in July (*p* < 0.0001) (Figure 1(d)). In addition, the culture isolation rate was inversely correlated with the *Ct* (Figure 1(e)) (*p* < 0.0001).

**Figure 1.**
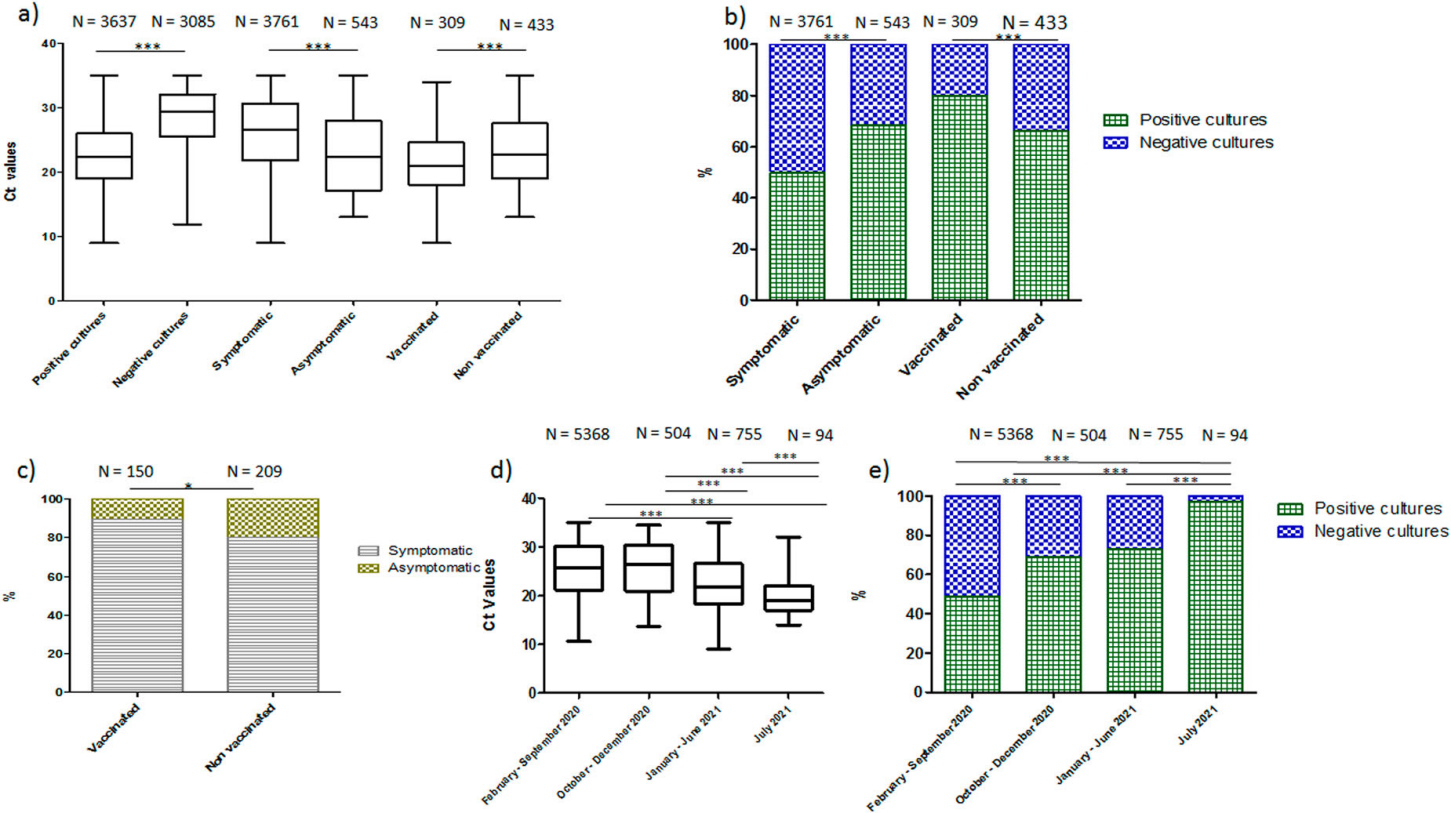
(a) Correlation between viral load evaluated by RT-PCR *Ct* and positive culture among symptomatics and asymptomatics patients, (b) percentage of negative and positive culture among symptomatic/asymptomatic and vaccinated/unvaccinated, (c) percentage of symptomatic patients among vaccinated and unvaccinated, (d) correlation between viral load evaluated by RT-PCR *Ct* and time of sampling, and (e) percentage of positive and negative cultures during time of sampling.

We herein confirm relationship between qPCR *Ct* at diagnosis and SARS-CoV-2 isolation despite culture viral isolation sensitivity could vary dramatically according to the procedure [1,2,5,6]. Such correlation was independent of the period of study, of the presence or absence of clinical symptoms, and of the vaccination status of the patients. Patients symptomatic at SARS-CoV-2 diagnosis had lower culture isolation rate than asymptomatic patients, regardless they were vaccinated or not (Figures 1(a–c)), probably because these later may test earlier in the course of infection, when viral loads are highest. Vaccinated patients have also higher culture isolation rates than unvaccinated (Figures 1(a,b)). A bias in the selection of patients coming to our institute to be tested for SARS-CoV-2 infection is possible, but such correlation has already been observed among healthcare workers immediately after vaccination for whom the absolute risk of testing SARS-CoV-2-positive was increased without obvious explanation [7]. As previously observed in London between November and December 2020, viral loads during the period for which the alpha variant largely predominated were higher than with previous variants [8]. The same picture is observed now with delta variant as compared to alpha variant in China [9] and US [10], regardless of their vaccination status. The lack of information about the proportion of fully vaccinated patients (2-dose course of vaccine ≥ 14 days before SARS-CoV-2 infection) could be considered a weakness in our work. After one dose of vaccine, specific antibodies against SARS-CoV-2 are produced within 2 weeks of injection, although rates of protection may vary from one patient to another so this is an important issue to consider [11]. However, *Ct* was also similar among samples from patients fully vaccinated or not in the study by Brown et al. [12] who enrolled 74% of patients fully vaccinated and 90% infected with the delta variant. The present work confirms that higher viral loads observed with the delta variant are correlated to a higher positivity rate of culture virus isolation, and therefore to higher contagiousness of patients, regardless of the vaccination status of the patients and/or the presence or absence of clinical symptoms.
